# The Role of the *IGF2* Methylation Score in Diagnosing Adrenocortical Tumors with Unclear Malignant Potential—Feasibility of Formalin-Fixed Paraffin-Embedded Tissue

**DOI:** 10.3390/biomedicines11072013

**Published:** 2023-07-17

**Authors:** Rebecca V. Steenaard, Richard A. Feelders, Fadime Dogan, Peter M. van Koetsveld, Sara G. Creemers, Madeleine H. T. Ettaieb, Folkert J. van Kemenade, Harm R. Haak, Leo J. Hofland

**Affiliations:** 1 Department of Internal Medicine, Division of Endocrinology, Erasmus Medical Center, 3015 CN Rotterdam, The Netherlands; 2 Department of Internal Medicine, Máxima MC, 5504 DB Veldhoven, The Netherlands; 3 CAPHRI School for Public Health and Primary Care, Ageing and Long-Term Care, Maastricht University, 6229 HX Maastricht, The Netherlands; 4 Department of Internal Medicine, Tergooi MC, 1213 XZ Hilversum, The Netherlands; 5 Department of Pathology, Erasmus Medical Center, 3015 CA Rotterdam, The Netherlands; 6 Department of Internal Medicine, Division of General Internal Medicine, Maastricht University Medical Centre+, 6229 HX Maastricht, The Netherlands

**Keywords:** adrenocortical carcinoma, adrenocortical adenoma, *IGF2*, methylation, formalin-fixed paraffin-embedded tissue

## Abstract

The differentiation between benign and malignant adrenocortical tumors based on pathological assessment can be difficult. We present a series of 17 patients with unclear malignant tumors, of whom six had recurrent or metastatic disease. The assessment of the methylation pattern of insulin-like growth factor 2 (*IGF2*) regulatory regions in fresh frozen material has shown to be valuable in determining the malignancy of adrenocortical tumors, although this has not been elaborately tested in unclear malignant tumors. Since fresh frozen tissue was only available in six of the patients, we determined the feasibility of using formalin-fixed paraffin-embedded (FFPE) tissue for this method. We isolated DNA from FFPE tissue and matched the fresh frozen tissue of three patients with adrenocortical carcinoma. Methylation patterns of *IGF2* regulatory regions were determined by pyrosequencing using different amounts of bisulfite-converted DNA (5 ng, 20 ng, 40 ng). Compared to fresh frozen tissue, FFPE tissue had a higher failure rate (fresh frozen 0%; FFPE 18.5%) and poor-to-moderate replicability (fresh frozen rho = 0.89–0.99, median variation 1.6%; FFPE rho = −0.09–0.85, median variation 7.7%). There was only a poor-to-moderate correlation between results from fresh frozen and FFPE tissue (rho = −0.28–0.70, median variation 13.2%). In conclusion, FFPE tissue is not suitable for determining the *IGF2* methylation score in patients with an unclear malignant adrenocortical tumor using the currently used method. We, therefore, recommend fresh frozen storage of resection material for diagnostic and biobank purposes.

## 1. Introduction

Adrenocortical tumors (ACT) can have various presentations, from small adrenocortical adenomas (ACA) needing no intervention to large adrenocortical carcinomas (ACC) with a high risk of recurrence or metastasis [1,2,3]. The determination of malignancy in adrenocortical tumors based on imaging and pathology is not always clear. An unenhanced CT scan of a homogenous tumor with <10 Hounsfield Units rules out ACC, while a large, inhomogeneous tumor with Hounsfield Units > 21 suggests ACC [4,5,6]. This leaves a group with an unclear diagnosis. The same problem occurs with pathological determination of tumor grade by means of the Weiss score. In patients with a Weiss score of 2–3 or with divergent characteristics, the diagnosis often remains uncertain. The Weiss score has the additional disadvantage of an imperfect inter-observer reliability [7]. Proper diagnosis of ACT is important for selecting the best treatment and duration of follow-up for the patient. For example, patients with a high risk of recurrence can benefit from adjuvant mitotane therapy, while for other patients, watchful waiting with CT imaging might be sufficient [1,2]. There is an unmet need for a more objective biomarker to determine malignant potential in ACT, especially for patients with unclear malignancy based on imaging and pathology alone [8].

Several molecular markers have been proposed as biomarkers for prognosis, such as steroid profile, differential gene expression, or methylation [9]. Insulin-like growth factor 2 (*IGF2*) is one of the growth factors involved in adrenocortical cell function. In normal cells, the *IGF2* gene is maternally imprinted and paternally expressed. Increased expression of *IGF2* is associated with malignancy, but expression levels are highly variable and, therefore, less suitable for clinical use. *IGF2* expression is regulated by the differential methylation of regulatory regions, including differentially methylated region 2 (*DMR2*), protein binding site *CTCF3* in the imprinting control region, and nearby gene *H19* [10]. This differential methylation has more stable patterns and can be transformed into an *IGF2* methylation score. The *IGF2* methylation score has shown to be valuable in the distinction between ACA and ACC [11,12]. In ACT with unclear malignant potential, the addition of the IGF2 methylation score might, therefore, improve the assessment of the tumor and may be useful for treatment decisions on adjuvant therapy and follow-up duration.

Before the IGF2 methylation score can be used as a diagnostic tool in clinical practice, further validation is needed in patients with ACT with unclear malignant potential. In this study, we will present a series of patients with unclear ACT and aim to explore the potential role of the IGF2 methylation score in their postoperative treatment and follow-up management. Thus far, the *IGF2* methylation score has only been validated in fresh frozen tissue, which is not widely available in clinical setting. We, therefore, aimed to determine whether formalin-fixed paraffin-embedded (FFPE) tissue is suitable to measure the *IGF2* methylation score.

## 2. Materials and Methods

We searched for patients with an unclear ACT in three different research databases. Inclusion criteria were adult patients with a localized ACT with unclear malignant potential as described in the patient record, diagnosed before 2016 to allow for sufficient follow-up. The unclear malignant potential was defined as a Weiss score of 2–3 or Weiss score of 0–1 with histopathological, biochemical, radiological, or clinical signs of malignancy. We selected six cases from the Dutch Adrenal Network retrospective cohort and 11 cases from two Dutch adrenal biobanks (Table 1) [13]. Electronic patient records were consulted for the pathology report, treatment modalities, and case history up to December 2020. In six of the selected patients, the IGF2 methylation score had previously been determined in stored fresh frozen tissue [11]. The *IGF2* methylation score is calculated from the mean, standard deviation score (SDS) from the methylation percentages of *CFTC3* CpGs 5–7, *DMR2* CpGs 2–4, and *H19* CpGs 1–3. A score below 1.28 indicates ACA; a score above 3.18 indicates ACC, and a score between 1.28 and 3.18 indicates an unknown ACT. The other patients had no available fresh frozen tissue. This study was conducted under the guidelines that had been approved by the Medical Ethics Committees of the Erasmus Medical Center and Máxima MC.

To explore whether FFPE tissue is suitable for determining the *IGF2* methylation score, we selected three patients with ACC. Each patient had stored FFPE tissue and matched fresh frozen tissue. DNA from fresh frozen tissue (1 section of 10 µm) was isolated using the Wizard Genomic DNA Purification Kit (Promega, Madison, WI, USA). DNA from FFPE tissue (1–3 sections of 10 µm) was isolated using the QIAamp DNA FFPE Tissue procedure (Qiagen, Hilden, Germany) following the manufacturer’s protocol. The isolated genomic DNA was sufficient for our downstream applications with a median concentration of 149 ng/µL (range 80–418 ng/µL) and a median 260/280 nm ratio of 1.84 (range 1.7–1.9). The DNA yield was optimal in our sample when using 2 sections of 10 µm FFPE tissue.

The isolated genomic DNA (1 µg) was converted with sodium bisulfite using the EpiTect Plus DNA Bisulfite Kit (Qiagen) following the manufacturer’s protocol. PCR amplification (FastStart High Fidelity PCR System kit, Roche, Basel, Switzerland) and pyrosequencing (PyroGold SQA reagent kit, Qiagen, on the Pyromark Q24 system) of the bisulfite converted DNA were performed as previously described using a predefined pyrosequencing assay of CpGs involved in expression of IGF2 (CTCF3 CpG 5–7, DMR2 CpG 2–4 and H19 CpG 1–3) [11]. This step was repeated six times with three different amounts of bisulfite-converted DNA (5 ng, 20 ng, and 40 ng, measured in two independent assays) to evaluate the failure rate and replicability of the assay and the correlation between FFPE and fresh frozen tissues. Pyrosequencing was considered a failure (not evaluable) when peaks were below or equal to H_2_O control. Statistical analysis was performed in SPSS version 26 and GraphPad Prism 8.2.1. Replicability and correlation were assessed using Spearman’s correlation coefficient (rho) because of non-parametric data.

## 3. Results

### 3.1. Case Series of ACT with Unclear Malignant Potential

We selected 17 patients with an unclear ACT, diagnosed between 2000 and 2016. All patients had localized disease with a Weiss score between 1 and 3 upon initial diagnosis (Table 1). The diagnosis was unclear for varying reasons, such as size, a positive PET-CT scan indicating high metabolic activity, hormone production, another classification system, and minor invasive behavior or necrosis.

During follow-up, six patients (cases 6, 7, 9, 12, 13, 14) developed recurrent disease or metastasis. In four patients, this occurred within 5 years. Two patients (cases 13, 14) had already been discharged from the follow-up and presented 7 and 17 years after diagnosis with physical complaints caused by pulmonary metastasis and local recurrence, respectively. Four patients (cases 6, 7, 9, 14) eventually died with metastases, and one patient (case 8) died from a cause unrelated to ACT. Five patients (cases 1, 7, 8, 10, 11) in our series received adjuvant mitotane therapy. Among these patients, one (case 7) developed metastatic disease within a year. This patient died from metastatic disease 4 years after initial diagnosis. The other four patients remained disease-free, with the follow-up ranging from 4 to 10 years.

We aimed to validate the use of the *IGF2* methylation score in our case series with unclear ACT. In six patients, the *IGF2* methylation score had previously been determined in stored fresh frozen tissue [11,12]. Five patients were classified as ACC and one as unclear according to the *IGF2* methylation score. Based on the score, one patient (case 1) was treated with adjuvant mitotane therapy and has been free of disease for 4 years. One patient (case 6) died with metastases 2 years after the diagnosis without adjuvant mitotane therapy. In this case, the *IGF2* methylation score was only determined after the development of metastasis. In hindsight, the addition of the *IGF2* methylation score at the time of diagnosis might have been beneficial in the decision-making process for this patient. The other four patients are currently free from recurrence or metastasis (range of follow-up: 2–8 years).

Based on these six patients alone, we cannot conclude whether the addition of the *IGF2* methylation score has a valuable role in the diagnosis of unclear ACT. Unfortunately, there was no fresh frozen tissue available from other patients. We, therefore, explored whether FFPE tissue is suitable for determining the IGF2 methylation score in these patients.

### 3.2. Feasibility of Using FFPE Tissue

We measured the methylation of *IGF2* regulatory regions *CTCF3*, *DMR2*, and *H19* in matched fresh frozen and FFPE tissues of three patients with ACC (Table S1). We discovered that the pyrosequencing failure rate was high in the FFPE tissues (10/54 FFPE assays; 0/54 fresh frozen assays). Nine of these failures occurred when using 5 ng and 20 ng DNA extracted from FFPE tissue, while the 40 ng assays only had one failure. None of the assays using fresh frozen tissue, including 5 ng DNA, showed any problems.

**Figure 1:**
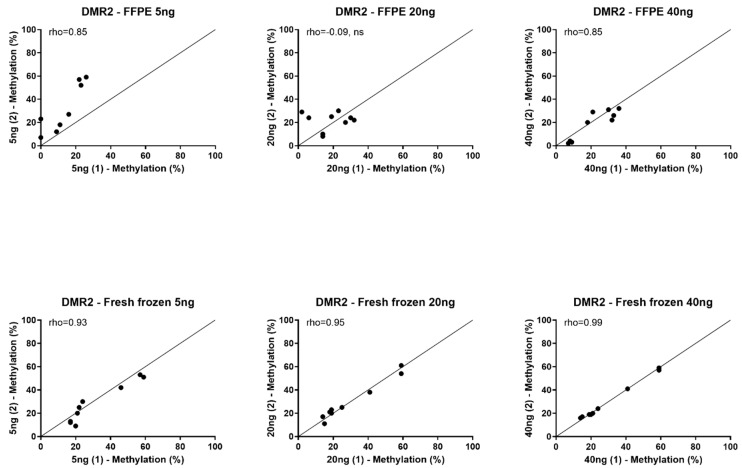
Replication of methylation percentage in FFPE and fresh frozen tissue. Spearman’s correlation (rho) of methylation percentages within formalin-fixed paraffin-embedded (FFPE) tissue replications (upper row) and fresh frozen tissue replications (bottom row). Pyrosequencing was performed using primers for *DMR2* CpG 2–4 in 5 ng, 20 ng, and 40 ng bisulfite-converted DNA, measured in two independent assays with the same concentrations of DNA. (1) first assay; (2) second assay. ns: not significant, *p* > 0.05. Reference line represents perfect replication.

Because of the high failure rate in FFPE-extracted DNA, replication of methylation percentages using the same amount of DNA could not be achieved for most assays. Only the DMR2 assays were completed in FFPE and fresh frozen tissues of all three patients without failures. In these assays, the replication in FFPE was poor-to-moderate (Spearman’s rho = −0.09–0.85) with a median variation of 7.7% in both directions (range 1–35%) (Figure 1). In contrast, replication of the methylation percentages from fresh frozen extracted DNA was very good across all assays (Spearman’s rho = 0.89–0.99) with low variation (median variation 1.6%, range 0–11%) (Figure 1 and Figure S1).

**Figure 2:**
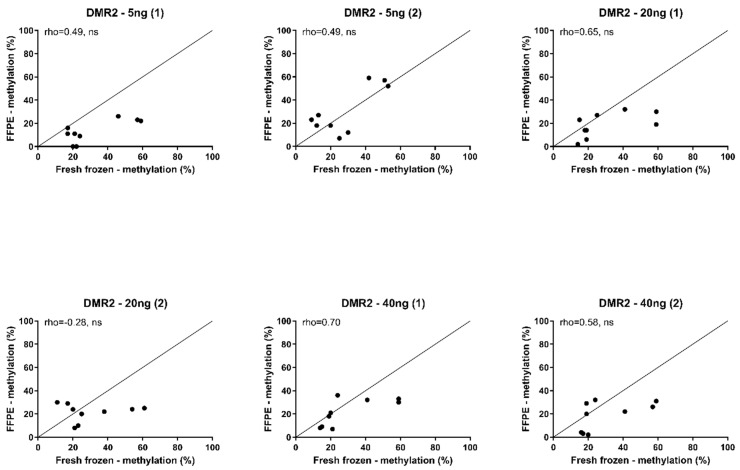
Correlation of methylation percentage in FFPE and fresh frozen tissue. Spearman’s correlation (rho) of methylation percentages between formalin-fixed paraffin-embedded (FFPE) tissue and fresh frozen tissue. Pyrosequencing was performed using primers for DMR2 CpG 2–4 in 5 ng, 20 ng, and 40 ng bisulfite-converted DNA, measured in two independent with the same concentrations of DNA assays. (1) first assay; (2) second assay. ns: not significant, *p* > 0.05. Reference line represents perfect replication.

We then compared the methylation percentages between the FFPE-extracted DNA to the matched fresh frozen tissues in the completed *DMR2* assays. We found the DNA methylation percentages from FFPE-extracted DNA to be poorly to moderately correlated to the percentages from fresh frozen tissues (Spearman’s rho = −0.28–0.70). The median variation between the methylation percentages found in FFPE and fresh frozen tissues was 13.2% in both directions (range 1–40%) (Figure 2).

## 4. Discussion

The determination of malignancy in ACT is not always clear, which can lead to difficult follow-up and treatment decisions. The current guidelines lack recommendations for the follow-up duration of patients with unclear malignant ACT [1,3]. Patients with ACA are discharged from follow-up after surgery, while patients with ACC are followed for at least 5 years, with a median time to recurrence of 11 months [14]. Many patients with unclear ACT will remain disease-free, and a long follow-up might result in unnecessary anxiety and costs [15,16]. However, our case series of 17 patients with unclear malignant ACT included four patients with recurrence within 5 years and two with late recurrence, suggesting that early discharge might result in underdiagnosis. Four patients eventually died from metastasis. In patients with proven ACC, adjuvant mitotane treatment is recommended in patients after radical surgery with a perceived high risk of recurrence (ENSAT stage III, R1 resection or Ki67-index > 10%). Adjuvant mitotane is, however, not recommended for unclear ACT [1]. Our case series included five patients with adjuvant mitotane therapy, of whom only one developed metastasis. Even though the series is small, these cases do suggest that watchful waiting might not always be the best option for patients with an unclear ACT. Some patients might benefit from adjuvant mitotane therapy; however, this decision is solely based on clinical judgment and patient preference. There is an unmet need for a more objective measure of malignancy and prognosis in patients with an unclear ACT to better guide the follow-up and treatment decisions.

The *IGF2* methylation score can help predict malignancy [11,12]. However, this has not yet been confirmed in a larger series of patients with unclear malignant ACT. We aimed to validate the use of the *IGF2* methylation score in our case series. Since only six patients had available fresh frozen tissue, and no conclusion could be drawn from their data alone, we determined the feasibility of using FFPE tissue in the other 11 patients. Unfortunately, we found a high pyrosequencing failure rate when using FFPE tissue (18.5%) and no failures when using fresh frozen tissue. Replication and correlation could, therefore, not be determined in most assays. Even in the completed *DMR2* assays, replicability in FFPE tissue was poor-to-moderate, while replicability in fresh frozen tissue was very good. These assays only had a poor-to-moderate correlation between *IGF2* methylation patterns in FFPE tissue and fresh frozen tissue of the same patients, and variations between the measurements were high in both directions. FFPE tissue is, therefore, not suitable for the determination of the *IGF2* methylation score in patients with unclear malignant ACT using the currently used laboratory method.

There have been several studies comparing the results of pyrosequencing methylation assays between FFPE and frozen tissue with varying results. Some studies show a promising correlation [17,18,19,20,21,22,23,24]. However, upon closer examination of the results, many studies experience high failure rates when using FFPE tissue [23,24,25,26,27], and both random and non-random deviations from the methylation percentage were found in frozen tissue [19,20,21,22,25,26,27,28,29]. Similar articles using other methods for methylation determination, such as Infinium Beadchip, Methylight, or methylation-specific PCR, show the same problems and limitations. Some studies find a good correlation between FFPE and frozen tissues [28,29,30,31,32,33], while others experience problems with failure rates [27,28,34,35,36] and poor correlation [28,29,36,37].

A possible explanation for the higher failure rate in FFPE tissue and the lack of replicability and correlation can be found in the degradation of DNA during storage [38]. DNA isolated from FFPE tissue can be of poor quality with high rates of fragmentation related to storage time and fixation protocol [39]. Compared to other methods for DNA isolation from FFPE tissues, the QIAmp method used in our study has a higher DNA yield in terms of quantity, quality, and PCR performance [40,41,42]. However, DNA in FFPE tissue already shows significant fragmentation before isolation. This fragmentation can lead to less successful PCR amplification and consecutive pyrosequencing failure, which we observed in our analysis. The use of short amplicons of less than 500 base pairs has, therefore, been recommended for analyzing DNA from FFPE tissue. Even though the amplicons in our study were less than 150 base pairs, we still experienced 18.5% pyrosequencing failure in the FFPE samples and no failures in the fresh frozen samples.

A second hypothesis is that the fresh frozen and FFPE tissue came from different parts of the same tumors. ACT have shown heterogeneity within one tumor [43]. We would expect this to impact the correlation between the results from FFPE tissue and fresh frozen tissue. However, we also found poor-to-moderate replicability when retesting the FFPE tissue, while the fresh frozen tissue resulted in near-perfect replication. This suggests another mechanism also causing random error, not only a systematic error due to heterogeneity.

A possible explanation for this random error is the introduction of DNA modifications during formalin fixation. These formaldehyde modifications can cause random errors during the bisulfite conversion [25]. Firstly, formalin fixation causes interstrand cross-linking resulting in incomplete conversion of unmethylated cytosines [22]. Secondly, mutational artifacts are introduced by hydrolytic deamination of methylated cytosines to thymines and unmethylated cytosines to uracil [44,45]. Methods for the removal of formalin modifications have been proposed over the years and applied in the QIAmp method for DNA isolation [22,45]. However, we still found more variation in the methylation levels measured in the FFPE tissue than in the fresh frozen tissue, suggesting random errors introduced in the analysis. The current method did not include a DNA-restauration step before Pyrosequencing. Further research should determine whether including this step in the process eliminates errors caused by formalin-fixation.

The *IGF2* methylation score is assessed in two independent cohorts to distinct ACA from ACC in fresh frozen tissue [11,12]. This can help to determine the duration of follow-up and whether or not to start adjuvant mitotane treatment. Ideally, we would like to validate this result for diagnostic use in patients with unclear malignant ACT. However, since stored FFPE tissues are not suitable, a prospective study should be performed with the collection of fresh frozen biobank material. The current guideline recommends operating on patients with suspected ACC in specialized adrenal centers [1]. Besides the higher chance of survival, these centers also have the capacity to handle and store fresh frozen tumor tissue [46]. We, therefore, recommend also operating on patients with unclear malignancy based on imaging in these specialized centers. In case of an unclear pathology result, a section of the fresh frozen tissue can then be sent to a central laboratory to perform the *IGF2* methylation analyses and determine the likelihood of malignancy. As an added advantage, these tissues can be stored in biobanks to facilitate future validation of the *IGF2* methylation score in unclear ACT and other research initiatives.

## 5. Conclusions

A subset of patients with unclear malignant ACT develops a recurrent adrenal tumor or metastases during (long-term) follow-up. Currently, there are no guidelines on how to monitor these patients and when to add adjuvant mitotane treatment. The *IGF2* methylation score is a candidate biomarker for prognosis in patients with ACT with unclear malignant potential and might aid follow-up and treatment decisions. Unfortunately, FFPE tissue is not suitable for determining the *IGF2* methylation score using the currently used method. We, therefore, recommend performing surgical resection of unclear ACT in specialized centers with the capacity for fresh frozen storage of pathological material for diagnostic and biobank purposes.

## Figures and Tables

**Table 1:** Disease history of cases.

Case	Diagnosis Pathologist	Diagnosis IGF2 Methylation Score	Treatment	History
1	Weiss 3, unclear malignancy, 3.8 cm, PET positive	3.51, ACC	Adjuvant mitotane	4 years disease-free
2	Weiss 3, adenoma, 10 cm, PET positive	3.34, ACC	Watchful waiting	4 years disease-free
3	Weiss 3, oncocytairy adenoma, 5.5 cm, PET positive high uptake	3.08, unclear	Watchful waiting	4 years disease-free
4	Weiss 1, 1 minor LWB criteria oncocytairy adenoma, 9.5 cm	4.29, ACC	Watchful waiting	2 years disease-free, discharged from follow-up
5	Weiss 1, partially invasive adenoma, 6 cm	4.20, ACC	Watchful waiting	8 years disease-free
6	Weiss 3	4.68, ACC	Watchful waiting	2-year death from positive lymph nodes and bone metastases
7	Weiss 3	No fresh frozen material	Adjuvant mitotane	1-year abdominal wall/omental metastases (resection); 2-year bone metastasis (RT/resection); 3-year lung metastasis (mitotane); 4-year death from disease
8	Weiss 3	No fresh frozen material	Adjuvant mitotane	10-years death from other cause, no disease recurrence
9	Weiss 3	No fresh frozen material	Watchful waiting	4-year death from peritonitis carcinomatosis
10	Weiss 2, cortisol and androgen production	No fresh frozen material	Adjuvant mitotane	12-years disease-free, discharged from follow-up
11	Weiss 3	No fresh frozen material	Adjuvant mitotane	9 years disease-free
12	Weiss 3	No fresh frozen material	Watchful waiting	3-year liver metastases (RFA/mitotane); 7-year recurrent liver metastases (resection/mitotane); 9 years disease-free
13	Adenoma with necrosis, 10 cm	No fresh frozen material	Watchful waiting	17-year recurrence 30 cm (resection); 20 years disease-free
14	Adenoma (upon revision after recurrence Weiss 4)	No fresh frozen material	Watchful waiting	7-year lung metastases (mitotane discontinued for liver toxicity, resection, RT); 10-year abdominal metastases (EDP); 12-year death from lung and abdominal disease
15	Weiss 3, 7.8 cm	No fresh frozen material	Watchful waiting	4 years disease-free
16	Weiss 2, 2.9 cm, cortisol production	No fresh frozen material	Watchful waiting	4 years disease-free, discharged from follow-up
17	Weiss 3, cortisol production	No fresh frozen material	Watchful waiting	4 years disease-free

ACC: adrenocortical carcinoma. EDP: etoposide, doxorubicin, cisplatin chemotherapy. PET: positron-emission tomography. RFA: radiofrequency ablation. RT: radiotherapy. IGF2 methylation score = mean SDS (*CFTC3*, *DMR2* and *H19*); <1.28 adrenocortical adenoma (ACA); >3.18 adrenocortical carcinoma (ACC); 1.28–3.18 unknown.

## Data Availability

The data presented in this study are available in Supplement Table S1.

## Supplementary Materials

The following supporting information can be downloaded at: https://www.mdpi.com/article/10.3390/biomedicines11072013/s1, Figure S1: Replication of methylation percentage in fresh frozen tissue *CTCF3*, *DMR2*, and *H19*; Table S1: Methylation percentage and *IGF2* methylation score of FFPE and fresh frozen tissues.
